# Friedberger and Fröhner’s Pathology and Therapeutics of the Domestic Animals

**Published:** 1895-04

**Authors:** 


### REVIEWS.

Friedberger and Fröhner's *Pathology and Therapeutics of the Domestic Animals.* Translated by Prof. W. L. Zuill. The second volume of this most excellent work has just reached us, covering the diseases of the organs of locomotion, nervous system, organs of respiration, chronic constitutional diseases, infectious and epizootic diseases. Like its predecessor, it is fraught with valuable information, stated in a clear and concise manner, added to which are many very valuable annotations from the French translation and from the pen of Prof. Zuill, to whom we owe a debt of gratitude for placing these volumes within the reach of English-speaking and reading veterinarians. When our libraries are enriched by such valuable additions it makes one feel that no longer is one's professional work hindered or unguarded by a valuable defence in time of need. The printer's art has been well exemplified in these volumes, and we sincerely trust that the sale of this work will be so gratifying that the author with others will feel justified in adding other German and French works to our English veterinary libraries.

